# Factors of accepting pain management decision support systems by nurse anesthetists

**DOI:** 10.1186/1472-6947-13-16

**Published:** 2013-01-29

**Authors:** Ju-Ling Hsiao, Wen-Chu Wu, Rai-Fu Chen

**Affiliations:** 1 Department of Hospital and Health Care Administration, Chia-Nan University of Pharmacy and Science, Tainan, Taiwan, Republic of China; 2 Department of Anesthesiology, Chi-Mei Medical Center, Tainan, Taiwan, Republic of China; 3 Department of Information Management, Chia-Nan University of Pharmacy and Science, No.60, Sec. 1, Erren Rd., Rende Dist, Tainan City, 71710, , Taiwan, Republic of China

**Keywords:** Pain Management, Decision support systems, Technology acceptance model, Anesthesiologist, Nurse-Anesthetist

## Abstract

**Background:**

Pain management is a critical but complex issue for the relief of acute pain, particularly for postoperative pain and severe pain in cancer patients. It also plays important roles in promoting quality of care. The introduction of pain management decision support systems (PM-DSS) is considered a potential solution for addressing the complex problems encountered in pain management. This study aims to investigate factors affecting acceptance of PM-DSS from a nurse anesthetist perspective.

**Methods:**

A questionnaire survey was conducted to collect data from nurse anesthetists in a case hospital. A total of 113 questionnaires were distributed, and 101 complete copies were returned, indicating a valid response rate of 89.3%. Collected data were analyzed by structure equation modeling using the partial least square tool.

**Results:**

The results show that perceived information quality (γ=.451, p<.001), computer self-efficacy (γ=.315, p<.01), and organizational structure (γ=.210, p<.05), both significantly impact nurse anesthetists’ perceived usefulness of PM-DSS. Information quality (γ=.267, p<.05) significantly impacts nurse anesthetists’ perceptions of PM-DSS ease of use. Furthermore, both perceived ease of use (β=.436, p<.001, R^2^=.487) and perceived usefulness (β=.443, p<.001, R^2^=.646) significantly affected nurse anesthetists’ PM-DSS acceptance (R^2^=.640). Thus, the critical role of information quality in the development of clinical decision support system is demonstrated.

**Conclusions:**

The findings of this study enable hospital managers to understand the important considerations for nurse anesthetists in accepting PM-DSS, particularly for the issues related to the improvement of information quality, perceived usefulness and perceived ease of use of the system. In addition, the results also provide useful suggestions for designers and implementers of PM-DSS in improving system development.

## Background

### Clinical decision support systems (CDSS) for nursing staffs

Nursing staff are the major group of healthcare professionals who perform crucial functions in delivering nursing care to inpatients. In addition, they work with patients and other caregivers and in collaboration with medical staff as members of multidisciplinary teams. Therefore, inappropriate or incompetent nursing actions endanger patient safety [1,2]. Traditionally, the duties of nursing personnel include (1) nursing assessments of health problems, (2) nursing measures for preventative healthcare, (3) nursing guidance and counseling, and (4) auxiliary medical care [3]. With the rapid changes in the delivery of healthcare, prior studies have indicated that nurses are undertaking extended roles, such as nurse practitioners (NP) and nurse anesthetists [3]. The introduction of clinical decision support systems (CDSS) is a possible method of supporting nurses within their extended roles and enhancing patient safety and quality of care [4-9]. Kawamoto et al. [10] showed that features of automatic provision for decision support as part of clinicians’ workflow, provision recommendations rather than only assessments, provision decision support at the time and location of decision making, and computer-based decision support are closely correlated with decision support systems’ abilities to significantly enhance patient care. Although there is scant research focusing on nurses’ use of CDSS compared with research focusing on doctors’ use of CDSS, some studies have revealed examples of CDSS in supporting nurses’ decision making in the management of angina [11] and diabetes [12], cancer pain [13], and triage for patients in first contact care [14,15].

### A need for pain management

Pain is regarded as the fifth vital sign in health assessment of patients’ statuses. Nurses, especially those with expertise in pain management, are valuable resources as health care organizations alter their pain assessment and management processes to meet pain standards [16]. Relieving pain during medical treatment is a common but critical healthcare issue encountered by healthcare institutions. However, meeting patient requirements for pain management is difficult because of individual differences regarding analgesic needs. To overcome the problem of traditional analgesia, the concept of pain management (pain control) or humanistic analgesia has been introduced to provide analgesia for postoperative pain in patients according to their needs, and to enable patients to participate in the analgesic process [9,17].

Prior study indicated that individualized pain management should take into account the onset, type, site, duration, intensity, and temporal patterns of the pain, concurrent medical conditions [18]. In addition, the study argued that the subjective perception of the intensity of pain that is not proportional to the type or to the extension of the tissue damage but depends on the interaction of physical, cultural, and emotional factors. Sun et al. [19] summarized the barriers to cancer pain assessment and management into three categories: patient, professional and system barriers. They found that lack of knowledge of the principles of pain relief, side effect management, or understanding of key concepts such as addiction, tolerance, dosing, and treatment of neuropathic pain are professional barriers affecting pain assessment and management. Thus, pain management is a critical and complex issue for anesthesiology.

The success of pain management depends on nurse anesthetists constantly monitoring patient statuses and making appropriate clinical assessments and analgesics based on patients’ statuses. Consequently, it is critical to provide a pain management decision support system (PM-DSS) for nurse anesthetists to assist in pain assessment, diagnosis, and intervention [12]. Randell et al. [20] found that nurses’ experience with the decision and the technology affected how they used a decision support system. They suggested that a nurses’ experience and their ability to adapt the technology to ‘fit’ their clinical practice is critical for the CDSS use.

### Pain management decision support systems (PM-DSS)

The pain management decision support system (PM-DSS), a CDSS and subsystem of healthcare or hospital information systems (HIS), provides decision support capabilities to healthcare professionals during patient pain management. Quinzio et al. [21] found that introducing an anesthesia information management system can enhance the quality of nurse anesthetists’ work. A PM-DSS can assist nurse anesthetists in collecting, storing, processing, acquiring, displaying, and transmitting data related to pain management. A prior study indicated that decision support systems for cancer pain management should include (1) a knowledge base generation module, (2) a decision-making module, and (3) a self-adaptation module [22]. Therefore, the PM-DSS developed in this study provides the following functions: (1) pain management services and information management during analgesic care (i.e., preoperative preparation, postoperative practice, and anesthetic practice); (2) provision of standardized patient care information during analgesic care; and (3) analysis of a pain management database to provide reference formulae for analgesia dosages suitable for patients’ physical conditions.

The PM-DSS records relevant aspects of pain management, ranging from patient admission to clinical care or hospice care units. Patient demographics and laboratory data are imported from the HIS and are supplemented with data that has been imported automatically from previous procedures and pain assessments. Data from respirators and vital-sign monitors are automatically gathered at defined intervals where compatible device interfaces are available [17].

### A need for understanding the acceptance of clinical decision support systems

Prior studies have found that users’ acceptance of information technology (IT) is crucial in determining whether IT promotion is successful [23,24]. Therefore, acceptance by healthcare professionals is essential for the successful adoption and implementation of healthcare-related systems [2,22-26]. Because the development of PM-DSS and CDSS in Taiwan remains in an early stage, an in-depth study must be conducted promptly to examine the factors that affect the successful development of PM-DSS in Taiwan. Randell and Dowding [27] found that clinician engagement is the critical element in the successful introduction of CDSS. Nurse anesthetists are crucial providers and operators in the processes of pain control and management, and their acceptance of PM-DSS is essential to success. When the PM-DSS is implemented, the opinions of nurse anesthetists must be the focus, concerning the extent to which the system helps them to integrate patient data rapidly and monitor patients' vital signs in a timely manner and enables patients to experience a safe and mild pain-control process.

This study investigated factors affecting PM-DSS acceptance from the perspective of nurse anesthetists using PM-DSS in a case hospital that implemented the system to support pain management decision processes. The research question of this study was “what critical factors affect nurse anesthetists’ PM-DSS acceptance?” The study results can help hospital managers understand the factors that affect PM-DSS use, thereby providing a reference when systems are introduced or promoted in the future.

The remainder of this paper is organized as follows. Methods describes the theoretical foundations, theoretical framework, instruments and participants, and methods for data analysis. Results shows the results. Discussion details an in-depth examination of the findings of this study. Finally, we address implications and offer a conclusion in Conclusions.

## Methods

### Theoretical foundations

The key to successful information systems lies in evaluating system acceptance from the users’ perspective [28]. The Technology Acceptance Model (TAM), originally proposed by Davis [29], is one of the most widely used theoretical models for predicting and explaining whether users will accept new IT or other systems [30]. Although the TAM has been applied to investigate factors affecting healthcare professionals’ acceptance of healthcare information technology (HIT) applications [31-38], inconsistent results have been found because of inherent differences between various user groups and application systems [24,33]. For example, research has shown that perceived usefulness and perceived ease of use may play a significant role in CDSS use among clinicians, but the influence of perceived ease of use is not significantly supported for other types of healthcare technologies [32]. Prior studies have found that TAM constructs are valid for healthcare professionals [2,25,26,38,39], but the perceived ease of use is not consistently related to attitudes [28,40].

Wu et al. [40] argued that TAM focuses more on technological aspect and its strengths are its parsimony and high explanatory power. They also indicated that TAM lacks consideration of the effects of human and organizational factors. Yarbrough and Smith [28] argued that one limitation of the TAM is its inability to consider the influence of external variables and barriers to technology acceptance. They suggested customizing the inclusion of variables to enhance the model’s accuracy. They further concluded that the major barriers to clinicians’ acceptance of systems can be classified into three major categories: human (personal) characteristics, organizational characteristics, and IS characteristics. In addition, Yusof et al. [38] proposed a human, organization, and technology-fit (HOT-fit) framework for evaluating the success of health information systems by emphasizing a good fit among human, organizational, and technical elements of the system. In their study, human factors included system use and user satisfaction; organizational factors included organization structure and environment; and technology factors included system quality, information quality, and service quality. These are considered the potential factors affecting PM-DSS acceptance.

Studies have been conducted to evaluate HIT from partial dimensions of the HOT-fit framework [27,41]. Randell and Dowding [27] emphasized organizational influences on nurses’ uses of CDSSs. They found that the key factors for the introduction of a CDSS are instigation from individual clinicians, initiatives at policy level, clinician engagement, the need for adequate resources, the characteristics of the system itself, and adequate training. Fitterer et al. [41] proposed a taxonomy for a multi-perspective assessment of HIT values in accordance with human and organizational considerations. They found system use to be related to people who use it, their level of use, their training, and their attitudes toward the system. Organizational factors consist of organizational structure, leadership, top management support, and medical staff sponsorship.

### Theoretical framework

**Figure 1:**
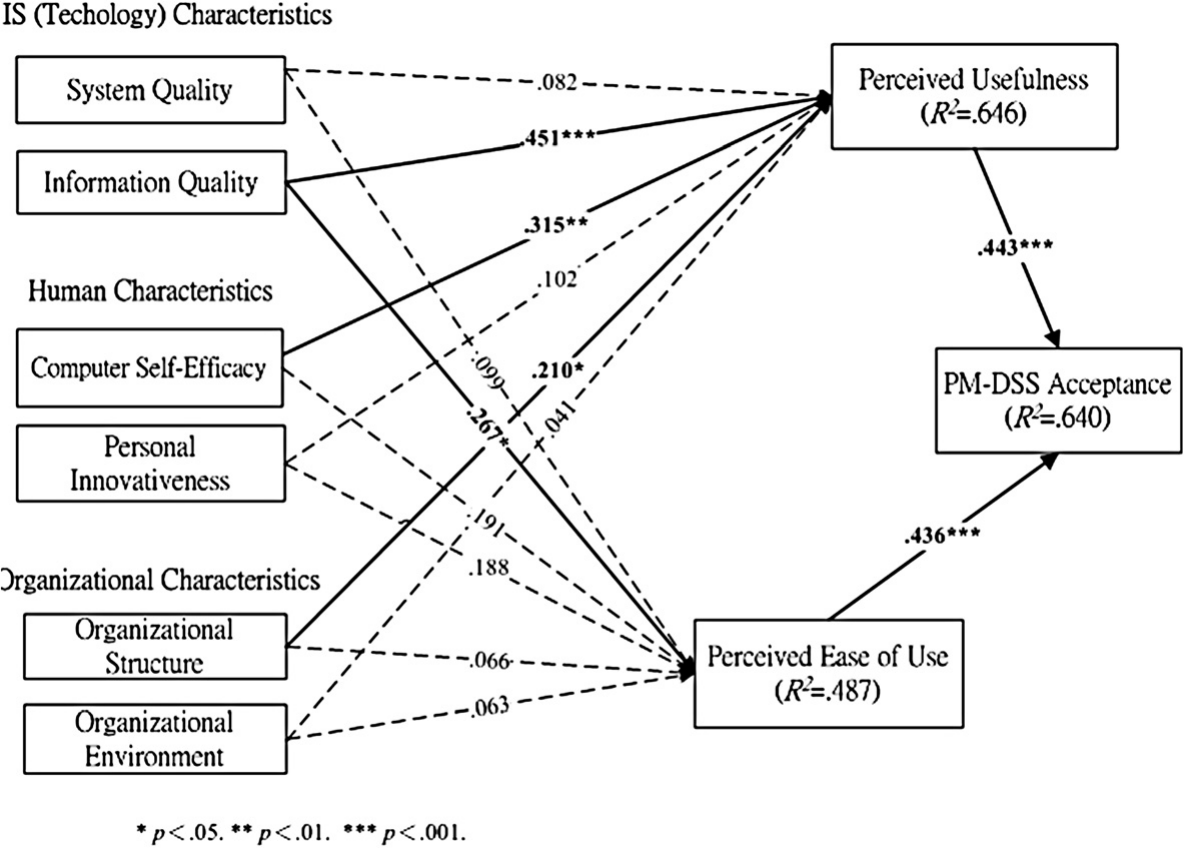
Result of model validity regarding factors affecting nurse-anesthetists PM-DSS acceptance.

To provide an in-depth investigation on factors affecting PM-DSS acceptance from the perspective of nurse anesthetists, we propose an extended TAM, as shown in Figure 1, derived from Davis’ original TAM framework [29] and the HOT-fit framework proposed by Yusof et al. [42]. The model emphasizes the influence of external variables and barriers to technology by incorporating the HOT-fit framework as the external factors of the TAM, as suggested by Yarbrough and Smith [28] and Yusof et al. [42]. These barrier factors could indirectly affect user attitudes and behavior intention toward IT use. Thus, the extended TAM consists of nine constructs: system quality, information quality, innovativeness, computer self-efficacy, organizational environment, organizational structure, perceived usefulness, perceived ease of use, and PM-DSS acceptance.

The IS barrier includes considerations of (1) system quality, that is, system response, system reliability, and security, and (2) information quality, meaning integrity, accuracy, format, completeness, and timeliness of information [43]. The development of a PM-DSS is a complex but important task for decision support on pain management in medical institutes. Therefore, measuring and evaluating the value and effectiveness of a PM-DSS is critical. Lu et al. [39] found that system quality and information quality are key factors influencing the perceived usefulness and perceived ease of use of an HIS. In addition, a previous study found that system quality and information quality are major factors affecting willingness and satisfaction in system use [44].

The human (personal) barrier includes (1) user self-efficacy (i.e., the ability of people to believe that they must apply IT systems to complete specific tasks) [45,46], and (2) personal innovativeness, representing the degree to which people are willing to take risks by test an innovation [47,48]. Prior research found that users are happier using IT when they have confidence in their ability to use computers [49]. Ong and Lai [50] found that users’ computer self-efficacy is a significant determinant of perceived usefulness and perceived ease of use in the context of e-learning. This finding implied that users with high computer self-efficacy are likely to have more positive usefulness and ease of use beliefs. In addition, past studies found that a higher level of personal innovativeness can lead to greater intention to use new technology [51], and a direct and positive correlation exists between personal innovativeness and personal perception of new technology benefits [52]. Accordingly, whereas healthcare professionals usually work in an independent decision-making manner for patient treatment and the early use of a PM-DSS is considered in a voluntary mode, personal innovativeness is argued as an important determinant of attitude toward using a PM-DSS.

The organizational barrier involves the following: (1) Organizational structure is a tool and instrument used by enterprises to achieve their objectives and to dominate and coordinate decision-making activities [53], and it may include type and size (number of beds), culture, politics, hierarchy, autonomy, planning and control systems, strategy, management and communications, leadership, top management support, and medical staff sponsorship [42], and (2) organizational environment, in the context of the environment in which the organization exists, includes external competition, influence of government policies, characteristics of target audience, and source of funds required for unit operations [42,54,55]. Kaplan [56] argued that the nature of a healthcare institution can be examined from its structure and environment. Yusof et al. [42] argued that organizational structure has an impact on system use, and they considered that organizational structure and organizational environment have influences on net benefits obtained from the use of an IS. Prior studies have found that external competition, influence of government policies, characteristics of target audience, and source of funds required for introducing new IS [42,54,55] are key factors for successful IT implementation. For example, patient safety initiatives and the promotion of electronic medical records (EMR) have led to an increased demand for PM-DSS development in hospitals in recent years [6,42].

Perceived usefulness represents users’ subjective beliefs in the benefits of using HIT to achieve job goals within medical practice [57]. When nurse anesthetists perceive a higher degree of system usefulness, they have more of a positive attitude and are willing to accept the PM-DSS. Perceived ease of use refers to the degree to which users believe that using HIT frees from effort [28]. Once users perceive that it is easier to learn how to use a system, they adopt a more positive attitude in accepting the system. According to the TAM, perceived usefulness and perceived ease of use both affect users’ attitude toward using IT, which consequently affects the actual behaviors of users [29]. Perceived ease of use also strengthens the users’ perception of the usefulness of IT; for example, if IT saves them time so that they can spend it on other tasks, this enhances their attitude toward IT use [28]. Huryk [58] found that the perception of enhanced patient care or safety and a system that was easy to use or integrated well into the nurses’ workflow were factors that lead toward a positive attitude. In addition, Aldosari [25] found that perceived ease of use and perceived usefulness are critical factors for user acceptance of picture archiving and communication systems (PACS). Satisfaction is often considered an important variable of IS success in the post-implementation stage [44]. In this study, we adopted satisfaction to measure system acceptance [59,60]. As discussed, we propose the following eight hypotheses:

H_1_: IS factors have a significant impact on nurse anesthetists’ perceived PM-DSS usefulness.

H_1a_: PM-DSS system quality affects nurse anesthetists’ perceived PM-DSS usefulness.

H_1b_: PM-DSS information quality affects nurse anesthetists’ perceived PM-DSS usefulness

H_2_: IS factors have a significant impact on nurse anesthetists’ perceived PM-DSS ease of use.

H_2a_: PM-DSS system quality affects nurse anesthetists’ perceived PM-DSS ease of use.

H_2b_: PM-DSS information quality affects nurse anesthetists’ perceived PM-DSS ease of use.

H_3_: Human factors significantly affect nurse anesthetists’ perceived PM-DSS usefulness.

H_3a_: Computer self-efficacy affects nurse anesthetists’ perceived PM-DSS usefulness.

H_3b_: Personal innovativeness affects nurse anesthetists’ perceived PM-DSS usefulness.

H_4_: Human factors significantly affect nurse anesthetists’ perceived PM-DSS ease of use.

H_4a_: Computer self-efficacy affects nurse anesthetists’ perceived PM-DSS ease of use.

H_4b_: Personal innovativeness affects nurse anesthetists’ perceived PM-DSS ease of use.

H_5_: Organizational factors significantly affect nurse anesthetists’ perceived PM-DSS usefulness.

H_5a_: Organizational structure affects nurse anesthetists’ perceived PM-DSS usefulness.

H_5b_: The organizational environment affects nurse anesthetists’ perceived PM-DSS usefulness.

H_6_: Organizational factors significantly affect nurse anesthetists’ perceived PM-DSS ease of use.

H_6a_: Organizational structure affects nurse anesthetists’ perceived PM-DSS ease of use.

H_6b_: The organizational environment affects nurse anesthetists’ perceived PM-DSS ease of use.

H_7_: Nurse anesthetists’ perceived PM-DSS ease of use affects system acceptance.

H_8_: Nurse anesthetists’ perceived PM-DSS usefulness of PMDSS affects system acceptance.

### Instrument and subjects

The initial research framework and questionnaires were developed through a literature review, and were revised by three experts in the anesthetic and medical-information field. These experts evaluated the content validity of the questionnaire, in which the measurement of expert validity is based on a content validity index (CVI) of 0.8 [61], and the overall CVI is 0.97, indicating excellent expert validity.

We collected empirical data from nurse anesthetists with more than one year of experience in interventional pain management. The case hospital was a private facility with 2100 beds, which had existed for over 40 years. The hospital is an early adopter in introducing innovative HIT in Taiwan, such as HIS, picture archiving and communication systems (PACS), and EMRs. The hospital is the earliest adopter in implementing mobile healthcare technology, namely mobile nursing information systems, to provide nursing staff with a more efficient manner in obtaining required information from HISs in a timely manner by using a portable information device and wireless technology. In 2010, the hospital established a PM-DSS to provide enhanced clinical diagnostic services and improve patient satisfaction. At present, the hospital has incorporated mobile healthcare technology into the PM-DSS to improve patient safety and quality of care. Nurse anesthetists were asked to use the PM-DSS in the processes of pain management. For these reasons, the hospital was selected as the case hospital in this study.

This study employed a survey methodology using a 39-item structured questionnaire, which was composed of two major parts: (1) recording the respondents’ demographic data, and (2) investigating factors affecting the acceptance of a PM-DSS. The questionnaire items were measured using a 5-point Likert scale, scored from 1 (*strongly disagree*) to 5 (*strongly agree*).

The IS construct included system quality and information quality, measured using eight items adapted from Otieno, Toyama, Asonuma, Kanai-Pak, and Naitoh [62] and Wixom and Todd [60]. The personal construct included user self-efficacy and innovativeness, measured using seven questions adapted from Vijayasarathy [49] and Agarwal and Karahanna [51]. The organizational construct addressed organizational structure and organizational environment, measured by 11 items adapted from Yusof et al. [42] and Sciulli [53]. Perceived usefulness was measured by eight items adapted from Wakefield et al. [57], and perceived ease of use was measured by three items adapted from Wixom and Todd [60]. PM-DSS acceptance was measured using two items adapted from Wixom and Todd [60]. Detailed descriptions of the questionnaire used in this study are provided in Table 1.

**Table 1:** Questionnaire

**Item no.**	**Item description**
1.SQ1	The PM-DSS is stable.
2.SQ2	The response time of PM-DSS is speedy.
3.SQ3	The data accessibility of PM-DSS is good.
4.SQ4	The effectiveness of PM-DSS security to prevent unauthorized access to patient data.
5.IQ1	The PM-DSS can integrate data from different sources.
6.IQ2	The information of PM-DSS is accurate.
7.IQ3	The content and its display format of PM-DSS can fulfill user needs.
8.IQ4	The information of PM-DSS is up-to-date.
9.IN1	If I heard that a new technology was available, I would be interested enough to test.
10.IN2	I prefer to use the most advanced technology available.
11.IN3	In general, I hesitate to try new information system.
12.CS1	I could complete the job using PM-DSS if I had never used a system like it before.
13.CS2	I could complete the job using PM-DSS if I had used similar system before PAIN MANAGEMENT DSS one to do the same job.
14.CS3	I have the ability to operate PM-DSS.
15.CS4	I prefer to use a PM-DSS for patient visit.
16.OS1	The employee should follow the clinical standard of procedures to complete clinical practice.
17.OS2	The employee could share his opinions with the supervisors and participate the decision processes in the pain management.
18.OS3	The duties and rights for the pain management were clarified in the work field and all were documented.
19.OS4	Greater degree of coordination achieved by grouping all those working on the pain management.
20.OS5	The clinical consultation problems would be resolved by many different ways.
21.OS6	There were champions for development of the PM-DSS.
22.OE1	The adoption of information technology in the hospital, which you serve, will be affected by medical policies.
23.OE2	The degree of competition among local hospitals is high.
24.OE3	The degree of computerization in our hospitals is high.
25.OE4	The requirement of patient care quality is high.
26.OE5	The organization provides enough funds to support the adoption of PM-DSS.
27.PU1	Using PM-DSS can reduce hospital patient care costs.
28.PU2	Using PM-DSS can improve work efficiency.
29.PU3	Using PM-DSS can improve patient care quality.
30.PU4	Using PM-DSS is helpful in assisting the collection and analyze of patient data.
31.PU5	Using PM-DSS can reduce the amount of time in paper work through PM-DSS.
32.PU6	Using PM-DSS can improve communication between physicians and hospital staff.
33.PU7	Using PM-DSS can improve patient safety.
34.PU8	Overall, PM-DSS is helpful in patient pain management.
35.PE1	Learning to use PM-DSS would be easy for me.
36.PE2	It would be easy for me to become skillful at using PM-DSS.
37.PE3	I would find it easy to get PM-DSS to do what I want it to do.
38.PA1	I am very satisfied with PM-DSS.
39.PA2	The PM-DSS functions perform as expected.

Note: SQ- System Quality; IQ- Information Quality; IN- Innovativeness; CS- Computer Self-Efficacy; OS- Organizational Structure; OE- Organizational Environment; PE- Perceived Ease of Use; PU- Perceived Usefulness; PA- PM-DSS Acceptance.

### Ethical considerations

To address potential ethical concerns, our study protocol and informed consent forms were reviewed and approved by the institutional review board (IRB) before the surveys were distributed and collected. After receiving approval from the IRB of the target hospital, research was conducted from mid-December to mid-January, 2011. Study participation was voluntary. Responses were anonymous and untraceable to individual nurses.

### Data analysis

The reliability and validity of the measurement model were assessed by confirmatory factor analysis (CFA) [63-65] by using the SmartPLS 2.0 software with the bootstrap resampling method (1000 resamples) to estimate the parameters of the research model. We used structural equation modeling (SEM) by employing the partial least squares (PLS) tool for data analysis [66] to examine the causal model.

## Results

### Demographic data

The survey was distributed to 113 nurse anesthetists of the case hospital, and 101 completed questionnaires were returned, indicating a valid response rate of 89.3%. This high response rate can be attributed to the top management support and voluntary participation of the respondents of the case hospital. All respondents were women. Most (91.0%) had a graduate diploma, and 83.1% had over 5 years of clinical practice experience. Most respondents (76.2%) were aged between 31 and 40 years. Almost all respondents (97%) have over 5 years of HIS experiences, and all respondents had more than 3 months of PM-DSS experience. Thus, the sample demographics showed that most respondents were experienced users in HISs and had rich experiences in clinical practice. The demographic data of the partcipants were shown in Table 2.

**Table 2:** Participant Demographic Data (N=101)

**Measure**	**Category**	**No(#)**	**Percent (%)**
Age	<30	7	6.9
	31-35	50	49.5
	36-40	27	26.7
	41-45	7	6.9
	46-50	9	9.0
	>50	1	1.0
Education level	Bachelor	91	91
	Master	9	9.0
Years of experience in clinical practice	<2	0	0.0
	2-5	7	6.9
	5-10	34	33.6
	>10	50	49.5
Years of experience in using HIS	<2	0	0.0
	2-5	2	2.0
	5-10	49	48.5
	>10	50	49.5
Experience in using PM-DSS	3-6 Months	60	59.4
	6-9 Months	20	19.8
	9-12Months	10	10.0
	>12Months	11	10.8

### Measurement model

Assessing the measurement model of this study involved testing the reliability, convergent validity, and discriminate validity [63]. The reliability and validity results of the research model are shown in Table 3. Constructs included reflective indicators and principal component analysis (PCA), provided by PLS, which ensured the unidimensionality of the constructs. PCA was used to determine that all indicators were significantly associated with only one latent variable, indicating the establishment of unidimensionality [67]. Composite reliability (CR) and average variance extraction (AVE) were used to evaluate reliability and convergent validity. The values of CR (>.810) and AVE (>.50) of all the constructs exceeded the recommended cutoff values of .7 and .5, representing good reliability and convergent validity. One criterion for adequate discriminant validity is that the square root of the AVE for each construct exceeds the correlation between the construct and other constructs in the research model [63]. All AVEs in this study were greater than the correlation coefficients, indicating good discriminant validity. As shown, this study had adequate reliability, convergent validity, and discriminant validity.

**Table 3:** Results of reliability and validity of the research model

			**Correlation matrix**									**AVE (≥0.5)**	**CR (≥0.7)**	**Cronbach’s α (≥0.7)**
	**Mean**	**SD**	**SQ**	**IQ**	**IN**	**CS**	**OS**	**OE**	**PU**	**PE**	**PA**			
SQ	**3.643**	**0.688**	1.000									0.743	0.930	0.885
IQ	**3.665**	**0.654**	0.644*	1.000								0.660	0.884	0.824
IN	**3.392**	**0.392**	0.387*	0.296*	1.000*							0.670	0.853	0.772
CS	**3.677**	**0.619**	0.372*	0.413*	0.728*	1.000						0.710	0.910	0.870
OS	**3.583**	**0.576**	0.485*	0.519*	0.290*	0.344*	1.000					0.567	0.886	0.844
OE	**3.573**	**0.488**	0.330*	0.421*	0.144*	0.315*	0.611*	1.000				0.501	0.810	0.715
PU	**3.733**	**0.594**	0.728*	0.726*	0.358*	0.540*	0.584*	0.468*	1.000			0.610	0.923	0.905
PE	**3.738**	**0.608**	0.501*	0.572*	0.490*	0.547*	0.439*	0.364*	0.653*	1.000		0.821	0.932	0.891
PA	**3.568**	**0.607**	0.501*	0.620*	0.385*	0.545*	0.530*	0.401*	0.728*	0.725*	1.000	0.925	0.961	0.919

SQ=System Quality; IQ=Information Quality; IN= Innovativeness; CS=Computer Self-Efficacy; OS=Organizational structure;

OE=Organizational environment; PU=Perceived usefulness; PE=Perceived ease of use; PA= PM-DSS Acceptance.

*p<0.001.

### Hypothesis testing

SEM was used to test the structural model and to enable examining the effects among the nine latent variables. As shown in Figure 1 and Table 4, six hypotheses were supported significantly in this study. Information quality (γ = .451, *p* < .001) in IS characteristics, computer self-efficacy (γ = .315, *p* < .01) in human characteristics, and organizational structure (γ = .210, *p* < .05) in organizational characteristics had a significant impact on nurse anesthetists’ perceptions of PM-DSS usefulness, supporting H_1b_, H_3a_, and H_5a_. Information quality (γ = .267, *p* < .05) in IS factors significantly affects nurse anesthetists’ perceptions of PM-DSS ease of use, supporting H_2b_. Finally, perceived ease of use (β = .436, *p* < .001, R^2^ = .487) and perceived usefulness (β = .443, *p* < .001, R^2^ = .646) significantly affected PM-DSS acceptance (R^2^ = .640), supporting H_7_ and H_8_. This implied that perceived ease of use and perceived usefulness account for 64% of the total explained variance in nurse anesthetists’ acceptance of PM-DSS. However, inconsistent with our hypotheses, the data show that system quality, personal innovativeness, and organizational environment have no significant impact on perceived usefulness (H_1a_, H_3b_, and H_5b_) and perceived ease of use (H_2a_, H_4b_, and H_6b_).

**Table 4:** Overall hypothesis validation results

**Hypothsis**	**Path coefficient**	**Result**
H_1_: IS factors have a significant impact on nurse anesthetists’ perceived PM-DSS usefulness.		**Partial support**
H_1a_: The system quality of the PM-DSS affects nurse anesthetists’ perceived PM-DSS usefulness.	0.082	No support
H_1b_: PM-DSS information quality affects nurse anesthetists’ perceived PM-DSS usefulness	0.451***	**Support**
H_2_: IS factors have a significant impact on nurse anesthetists’ perceived PM-DSS usefulness.		**Partial support**
H_2a_: The system quality of the PM-DSS affects nurse anesthetists’ perceived PM-DSS ease of use.	0.099	No support
H_2b_: PM-DSS information quality affects nurse anesthetists’ perceived PM-DSS ease of use.	0.267*	**Support**
H_3_: Human factors significantly affect nurse anesthetists’ perceived PM-DSS usefulness.		**Partial support**
H_3a_: Computer self-efficacy affects nurse anesthetists’ perceived PM-DSS usefulness.	0.315**	**Support**
H_3b_: Personal innovativeness affects nurse anesthetists’ perceived PM-DSS usefulness.	0.102	No support
H_4_: Human factors significantly affect nurse anesthetists’ perceived PM-DSS ease of use.		No support
H_4a_: Computer self-efficacy affects nurse anesthetists’ perceived PM-DSS ease of use.	0.191	No support
H_4b_: Personal innovativeness affects nurse anesthetists’ perceived PM-DSS ease of use.	0.188	No support
H_5_: Organizational factors significantly affect nurse anesthetists’ perceived PM-DSS usefulness.		**Partial support**
H_5a_: Organizational structure affects nurse anesthetists’ perceived PM-DSS usefulness.	0.210*	**Support**
H_5b_: The organizational affects nurse anesthetists’ perceived PM-DSS usefulness.	0.041	No support
H_6_: Organizational factors significantly affect nurse anesthetists’ perceived PM-DSS ease of use.		No support
H_6a_: Organizational structure affects nurse anesthetists’ perceived PM-DSS ease of use.	0.066	No support
H_6b_: The organizational affects nurse anesthetists’ perceived PM-DSS ease of use.	0.063	No support
H_7_: Nurse anesthetists’ perceived PM-DSS ease of use affects system acceptance.	0.436***	**Support**
H_8_: Nurse anesthetists’ perceived PM-DSS usefulness of PMDSS affects system acceptance.	0.443***	**Support**

*p<0.05. **p<0.01. ***p<0.001.

## Discussion

This study proposed an extended TAM by incorporating Davis’ original TAM framework [29] and the HOT-fit framework of Yusof et al. [42] to investigate the critical factors affecting PM-DSS acceptance from nurse anesthetists’ perspectives. The major findings of this study included that (1) information quality significantly affects the perceived ease of use of PM-DSS; (2) information quality, organizational structure, and computer self-efficacy significantly influence the perceived usefulness of PM-DSS; and (3) perceived ease of use and perceived usefulness influence PM-DSS acceptance by nurse anesthetists. The data showed that perceived usefulness has a substantial influence that is slightly greater than that of perceived ease of use on PM-DSS acceptance by nurse anesthetists.

### Factors affecting nurse anesthetists’ perceived ease of use and perceived usefulness

Consistent with findings obtained by Lu et al. [39], the results of this study show that perceived information quality has the most significant impact on perceived ease of use. Information quality involves users’ evaluations of the integrity, accuracy, format, completeness, and timeliness of a PM-DSS. Prior studies have argued that the quality of information is critical for CDSS development [68-71]. This can explain the critical role that the information quality of a PM-DSS plays for nurse anesthetists’ perceived ease of use of a PM-DSS.

The results show that perceived information quality, computer self-efficacy, and organizational structure all have a significant impact on nurse anesthetists’ perceived usefulness of a PM-DSS. The most significant influences on nurse anesthetists’ perceived usefulness of a PM-DSS were, in rank order, perceived information quality, computer self-efficacy, and organizational structure. We found perceived information quality to be a significant factor influencing PM-DSS perceived usefulness. Our findings also indicated a consistent result with prior CDSS-related studies [58,71].

Because the perceived information quality generated by a PM-DSS has a considerable influence on clinical decision making, nurse anesthetists need high information quality when providing clinical, medical, and care services for patients. Thus, PM-DSS development should consider the capacities of a PM-DSS to integrate data from different sources; system accuracy, timeliness, and completeness of generated information; and the degree of compliance of information display methods and definition with professional requirements [57]. Thus, hospitals can obtain anticipated benefits of PM-DSS use when the system is well-designed and implemented according to the unique clinician requirements of patient safety. It can help enhance PM-DSS perceptions among medical staff [72].

User self-efficacy refers to the ability that people have in believing that they must apply IT systems to complete specific tasks [46]. Consistent with the results obtained by Ong and Lai [50] and Wu et al. [2], we found perceived user self-efficacy to be a significant factor affecting PM-DSS perceived usefulness. Yi and Hwang [73] found behavior modeling training and observational learning processes to have been linked to increased self-efficacy in the context of computer training. Quinzio et al. [21] argued that the perceived quality of training strongly influenced user acceptance of an anaesthesia information management system. Huryk [58] found that increased computer experience is the key indicator for positive attitudes. Thus, hospitals should provide adequate technical support and training and incentives to promote and facilitate the PM-DSS use of nurse anesthetists, to increase the expected benefits obtained from system use.

Organizational structure is a tool used by enterprises to achieve their objectives and to dominate and coordinate decision-making activities [53]. Consistent with Yusof et al. [42] and Lluch [74], we found that perceived organizational structure is a critical factor affecting nurse anesthetists’ perceived PM-DSS usefulness. Nursing staff members are key collectors, generators, and users of patient/client information in healthcare management. Anesthetic practice is a professional specialty that requires communication and coordination between anesthesiologists and nurse anesthetists with varying professional knowledge and skills to increase the quality of care and patient safety during pain management. A PM-DSS is a complex IS that nurse anesthetists use to monitor, record, and store information on analgesia use. When the PM-DSS is designed and implemented according to the standardized clinical procedures and processes in pain management, the system can fulfill the information needs of nurse anesthetists and can further improve the perceived usefulness of PM-DSS.

Although several critical factors affecting PM-DSS acceptance by nurse anesthetists have been identified, perceived system quality, personal innovativeness, and organizational environment were found to have no significant effect on perceived ease of use and perceived usefulness of a PM-DSS. System quality includes the characteristics of system response, system reliability, and security. Contrary to the findings of prior studies [26,39], the relationships between perceived system quality and perceived ease of use and perceived usefulness were not significantly supported in this study. A PM-DSS is actually a typical CDSS and a subsystem of HIS. As mentioned, CDSS-related studies [68-70] and HIS-related study [75] focused more on perceived information quality than system quality. Consistent with our study, Hsiao et al. [75] found that system quality is not a significant factor for nurses’ perceived usefulness and perceived ease of use of HIS. This may account for the insignificance of perceived system quality on perceived ease of use and perceived usefulness of a PM-DSS.

Personal innovativeness represents the degree to which a person is willing to risk testing an innovation [47,48]. A prior study found that perceived personal innovativeness is not a significant factor affecting nursing acceptance of a decision support computer program for cancer pain management [13], which is a result that is consistent with those of our study. A possible explanation may be that the mandatory use of a PM-DSS in the case hospital may impede nurse anesthetists’ willingness to risk testing the PM-DSS.

An organizational environment is the environment in which an organization exists, including external competition, influence of government policies, characteristics of the target audience, and source of funds required for unit operations [42,54,55]. Regarding government policy, nurse anesthetists believe it to be important; thus, no statistically significant difference existed between the nurse anesthetists for this factor. However, if the government requires them to have a PM-DSS, all hospitals agreed that they would begin immediate adoption. Therefore, if the Taiwanese government and state-run insurance agencies implement such a requirement, all hospitals would be forced to comply, irrespective of whether they are ready to do so. In addition, nurses have a firm grasp of their own domains, and they have the authority to delegate MIS tasks to other departments. This separation of duties has contributed to a lack of efficient communication between specialists, and underscores the need for IT facilitators/consultants intimate with both nursing and MIS realities. Considering that IT literacy among nurses is critical and that hospitals may be required by the government to adopt PM-DSSs in the future, nursing specialists who possess a high degree of knowledge and experience with IT/MIS can play a pivotal role as consultants and project facilitators. These are possible reasons for explaining the non-significance of the organizational environment in this study.

### Relationships between perceived ease of use, perceived usefulness and PM-DSS acceptance

The study results show that perceive ease of use and perceived usefulness have a significant impact on PM-DSS acceptance. This implied that, first, PM-DSS acceptance by nurse anesthetists is affected mainly by their perceptions of perceived usefulness, and second, by their perceptions of the perceived ease of use. The findings are consistent with the results of prior studies that investigated the acceptance of systems from the perspectives of health care professionals [25,26,31,32,39,76,77]. Healthcare professionals are more pragmatic, and they should focus more on the usefulness and ease of use of HIT [78]. Nurse anesthetists are likely to accept and use PM-DSSs when they are considered useful and ease to use in their clinical practice related to pain management. A prior TAM study also suggested that perceived usefulness and perceived ease of use of an IT/IS determine the attitudes of people related to IT/IS [29]. Nurse anesthetists are more accepting toward PM-DSS when they have a positive attitude toward it. Thus, perceived usefulness and perceive ease of use are important factors in PM-DSS acceptance from the perspective of nurse anesthetists.

### Strengths and limitations of the research

Pain management is a critical but complex issue in the relief of acute pain, particularly for postoperative and severe pain in cancer patients. The introduction of a pain management-decision support system is critical for nurse anesthetists in assisting pain assessment, diagnosis, and intervention. This investigates factors affecting acceptance of PM-DSS from nurse anesthetists’ perspectives. Compared with TAM-based research, our proposed extended TAM model demonstrated a highly totally explained variance better than do traditional TAM (40%) or TAM2 (59%) studies [79]. In addition, all respondents were experienced users in HIS and PM-DSS. Therefore, the results of this study can be extended to other healthcare professionals with similar levels of experience. The results of this study enable hospital managers to understand the important considerations for nurse anesthetists in accepting PM-DSS, particularly for issues related to enhancing information quality, perceived usefulness, and perceived ease of use of the system. In addition, this study provides useful suggestions for designers and implementers of PM-DSS in further system development.

The findings of this study are subject to three major limitations. First, the study was performed in a single medical center hospital because the development of PM-DSS in Taiwan is in an early stage. In addition, the data derived from questionnaires was provided by participants with more than 3 months of experience using PM-DSS. Respondents answered questions based on their perceptions, experiences, and understandings. Consequently, the data collected may not be adequately objective. Therefore, the respondents may not sufficiently represent the nurse anesthetist’ population, inhibiting the generalization of this study. Second, we only tested cross-sectional data collected by nurse anesthetists in one period. Finally, the use of PM-DSS in the case hospital is mandatory. Therefore, the findings of this study should be carefully evaluated when applied to a context of voluntary use.

## Conclusions

This study proposed and validated an extended TAM derived from Davis’ original TAM framework and the HOT-fit framework proposed by Yusof et al. to investigate the factors affecting PM-DSS acceptance by nurse anesthetists. The findings showed that nurse anesthetists’ PM-DSS acceptance is substantially affected by the perceived usefulness and perceived of use of the system, which is consistent with the results of most TAM studies. In addition, the results showed that perceived information quality is the most significant factor affecting perceived ease of use and perceived usefulness. In addition, nurse anesthetists’ computer self-efficacy and organizational structure affected PM-DSS acceptance in this study. Through this study, factors influencing nurse anesthetists’ PM-DSS acceptance were identified, which can help hospital managers devise appropriate strategies in the early stages of system development when healthcare ISs are introduced.

This study demonstrates the critical role of perceived information quality on the successful implementation of PM-DSS, and the results are consistent with prior CDSS studies [68-71] in that the perceived information quality of a decision support system can influence subsequent decision quality and decision performance. Therefore, the design and implementation of a CDSS, similar to a PM-DSS, should focus on understanding and collecting the informational needs of nurse anesthetists and stakeholders related to PM-DSSs to deliver data with integrity, accuracy, format, completeness, and timeliness for decision making in pain management. Furthermore, hospitals should establish a supportive environment for PM-DSS development and encourage nurse anesthetists and other stakeholders to jointly participate in system design and implementation. Technical support and training should be provided to major users of PM-DSSs to reduce user resistance to PM-DSSs, enhance the computer self-efficacy of nurse anesthetists, and facilitate DSS use. Hospitals should focus on nurse anesthetists’ suggestions and feedback during PM-DSS development and implementation to obtain necessary information for system enhancement.

## Competing interests

The author declares that they have no competing interests.

## Authors’ contributions

J-LH is the first author of this paper. She took the responsibilities for the conception and design of the study and collection, analysis and interpretation of data. She also helped to compile the submitted paper and made required modifications of the paper. W-CW is the second author of this paper. She is an expert in nurse-anesthetist with over twenty-year experience. She provided assistances in research design, data collection and interpretation. R-FC is the corresponding author of this paper. He helped to propose adequate research concepts and strategies of this study. He also provided useful suggestions in research design and the collection, analysis and interpretation of data. All authors read and approved the final manuscript.

## Authors’ information

Ju-Ling Hsiao is an assistant professor of department of Hospital and Health Care Administration at the Chia-Nan University of Pharmacy and Science. Her research interests include nursing informatics, electronic medical record, and hospital information systems. Her published works have appeared in *CIN-Computers, Informatics, Nursing*, *International Journal of Medical Informatics*, *Journal of Nursing Research*, *Telemedicine and e-Health*, and *Total Quality Management & Business Excellence*.

Wen-Chu Wu completed his MS degree in Hospital and Health Care Administration at the Chia-Nan University of Pharmacy and Science. She is a nurse anesthetist and have worked as nursing administrator in a medical center for the past twenty years. Her research interests include studies of pain management, nursing informatics, and hospital information systems.

Rai-Fu Chen is an assistant professor of department of Information Management at the Chia-Nan University of Pharmacy and Science. His research interests include medical informatics, electronic medical record, and healthcare information systems. His published works have appeared in *CIN-Computers, Informatics, Nursing*, *International Journal of Medical Informatics*, *Journal of Nursing Research*, *Telemedicine and e-Health*, and *Total Quality Management & Business Excellence*.

## Pre-publication history

The pre-publication history for this paper can be accessed here:

http://www.biomedcentral.com/1472-6947/13/16/prepub
